# Partial Discharge Spectral Characterization in HF, VHF and UHF Bands Using Particle Swarm Optimization

**DOI:** 10.3390/s18030746

**Published:** 2018-03-01

**Authors:** Guillermo Robles, José Manuel Fresno, Juan Manuel Martínez-Tarifa, Jorge Alfredo Ardila-Rey, Emilio Parrado-Hernández

**Affiliations:** 1 Department of Electrical Engineering, Universidad Carlos III de Madrid, Leganés, 28911 Madrid, Spain; jfresno@ing.uc3m.es (J.M.F.); jmmtarif@ing.uc3m.es (J.M.M.-T.); 2 Department of Electrical Engineering, Universidad Técnica Federico Santa María, 8940000 Santiago de Chile, Chile; jorge.ardila@usm.cl; 3 Department of Signal Processing and Communications, Universidad Carlos III de Madrid, Leganés, 28911 Madrid, Spain; eparrado@ing.uc3m.es

**Keywords:** partial discharges, measurements in UHF, dimensionality reduction methods, particle swarm optimization, spectral analysis, signal characterization

## Abstract

The measurement of partial discharge (PD) signals in the radio frequency (RF) range has gained popularity among utilities and specialized monitoring companies in recent years. Unfortunately, in most of the occasions the data are hidden by noise and coupled interferences that hinder their interpretation and renders them useless especially in acquisition systems in the ultra high frequency (UHF) band where the signals of interest are weak. This paper is focused on a method that uses a selective spectral signal characterization to feature each signal, type of partial discharge or interferences/noise, with the power contained in the most representative frequency bands. The technique can be considered as a dimensionality reduction problem where all the energy information contained in the frequency components is condensed in a reduced number of UHF or high frequency (HF) and very high frequency (VHF) bands. In general, dimensionality reduction methods make the interpretation of results a difficult task because the inherent physical nature of the signal is lost in the process. The proposed selective spectral characterization is a preprocessing tool that facilitates further main processing. The starting point is a clustering of signals that could form the core of a PD monitoring system. Therefore, the dimensionality reduction technique should discover the best frequency bands to enhance the affinity between signals in the same cluster and the differences between signals in different clusters. This is done maximizing the minimum Mahalanobis distance between clusters using particle swarm optimization (PSO). The tool is tested with three sets of experimental signals to demonstrate its capabilities in separating noise and PDs with low signal-to-noise ratio and separating different types of partial discharges measured in the UHF and HF/VHF bands.

## 1. Introduction

The measurement of partial discharges (PDs) is a powerful and flexible technique to monitor and detect on-line advanced ageing in all types of high-voltage equipment [1,2]. A step forward for these on-line measurements is the use of the radiation emitted by PD sources by antennas tuned in the band of frequencies of the emitters, a technique widely used mainly in open-air substations [3,4]. Detecting the pulses also allows to locate geometrically the defects using multilateration techniques based on the time differences of arrival (TDOA) [5] or other methods based on the received signal strength (RSS) [6] or the angle of arrival (AOA) [7]. However, PD signals acquired in the radio-frequency (RF) range usually have magnitudes much lower than those obtained with other techniques which, together with the noise received from interferences in the same band of frequencies leads to great difficulties in the identification of the PD. This paper proposes a tool that is able to assign the received signals to a certain cluster, and then separates automatically the clusters in a classification map based on fingerprints present in the frequency spectrum using particle swarm optimization (PSO).

Signal representation in the frequency domain is key in the solution of most signal processing problems due to the fact that the spectrum of signals is strongly related to their source and nature. This is especially important in the measurement of partial discharges with sensors in the ultra high frequency (UHF) range since the path followed by the emission imprints a signature in the signal that can be used to classify and separate different types of events. In general, signal characterization [8], facilitates greatly the processing reducing computational burden (the representation of the acquired signal as a sequence of frequency or time samples is replaced by a few scalars) and simplifies the interpretation and analysis of the results by humans. The focus of this work is on the identification of PDs through a selective spectral characterization representing each signal with the energy contained in the *n* most informative UHF or high frequency (HF) and very high frequency (VHF) bands. Specifically, n=2 in this paper since we are interested in the design of visualization tools based on scatter plots, heat maps, etc. Such simple signal characterization will certainly increase the usability of the corresponding systems. Our starting point for the design of the procedure is a set of signals represented in terms of their spectrum. Each sampled partial discharge or each interference spectra can be regarded as a datum, formed by *m* features: the value of the power spectral density in the corresponding frequency. The selective spectral characterization can thus be considered as a dimensionality reduction problem: transform each *m* dimensional spectrum in an *n* dimensional array in which each component is the energy contained in one of the frequency bands of interest.

Dimensionality reduction techniques, [9,10], have been long used in machine learning. These techniques can lead to improvements in the performance of general purpose machine learning algorithms along three axes:

- improvements in accuracy due to the remotion of noisy or irrelevant information from the observations,
- improvements in the numerical stability of algorithms due to the remotion of redundant features, and
- facilitating the visualization and interpretation of the results.

Dimensionality reduction methods are grouped into two main families: feature selection and feature extraction. On the one hand, feature selection methods remove redundant and irrelevant features to yield the minimal subset of the original features that contains the information necessary for solving the problem at hand. Broadly used feature selection methods are Lasso [11,12] or Recursive Feature Selection [13]. On the other hand, feature extraction techniques transform the initial set of variables in a new, reduced set in a way that the new variables contain only relevant information. Principal Component Analysis [14,15], Orthogonal Partial Least Squares [16] or t-Stochastic Neighbors Embedding [17] are widely used examples of feature extraction techniques.

A big problem with these dimensionality reduction methods is that they would obscure the interpretation of the results of the processing. Feature selection techniques would come up with sets of scattered frequencies, not necessarily forming meaningful bands since, in most scenarios, adjacent frequencies will be highly correlated and the feature selection method would filter out correlated features. In the case of feature extraction, each resulting new feature comes from a transformation that merges and melts the original frequencies. This greatly hampers the determination, the relevance and the influence of each frequency band in the final result.

As introduced before, the selective spectral characterization is a preprocessing tool that facilitates the main processing. This paper is focused on a clustering of signals that could form the core of a visual PD monitoring system. Since the clustering is performed in the frequency domain, our work relates to feature-based clustering approaches according to the taxonomies for signal clustering presented in [18]. Therefore, the dimensionality reduction technique should determine the best frequency bands to enhance the similarities between signals in the same cluster and the differences between signals in different clusters.

This paper proposes a novel approach that interleaves the selective spectral characterization with the clustering in a same optimization without an a priori knowledge of the spectral power distribution in the signals. This is particularly interesting in the case of the UHF detection of partial discharges since their spectra depend on uncontrollable factors such as the discharging site, reflections, line-of-sight and interferences from radio, TV broadcasting and mobile communications. The joint optimization alternates between an optimization with metaheuristics that refines the frequency bands that support the signal characterization and the optimization of the clustering criterion using the signals characterized with these bands as dataset.

The capabilities of the method are illustrated in several experiments involving the detection and classification of PDs in high-voltage equipment.

The remainder of the paper is organized as follows: Section 2 explains the process to extract the spectral information from signals reducing the information of separability to clusters in two dimensions for the sake of clearness in the interpretation of the results. Section 3 justifies the criterion defined to maximize the distance between clusters considering the scattering in the clouds and the number of clusters. Section 4 describes the particle swarm optimization process to maximize the distance function proposed in the former section and sets the constraints to be accomplished in the clustering process. Afterwards, Section 5 shows the performance of the method in three experiments involving the separation of PDs emitted by two different sections in a cable with the aim of localizing the sources, the separation of lowsignal-to-noise ratio (SNR) PD and UHF interferences and the separation of three types of partial discharges in the HF/VHF range. Finally, Section 6 draws the main conclusions of the work.

## 2. Spectral Power Maps

This technique is applied to separate signals corresponding to different events characterizing them through their spectral power and finding those bands of frequency where their spectra is different. The study done in this paper is based on two bands of frequency because the representation is very intuitive in a two dimensional map; however, the extension of the algorithm to *n* dimensions is straightforward.

Let $f_{1L}$ and $f_{2L}$ be the start and end frequencies, respectively, for the first band and $f_{1H}$ and $f_{2H}$ the extremes of the second band. The subindex *L* states that the interval is placed at lower frequencies than the second band which has the subindex *H* for higher frequencies. The significant parameter of the signals is the spectral power calculated in those frequency bands referred to the total power of the signal, so low-energy signals have the same importance in the process as high-energy ones. Then, every signal would be parameterized with a power ratio at low frequencies, or PRL, and a power ratio at high frequencies, PRH:

(1) $$\begin{array}{ccc} PRL & = & \frac{\sum_{f=f_{1L}}^{f_{2L}}{\left|G(f)\right|}^{2}}{\sum_{f=0}^{f_{T}}{\left|G(f)\right|}^{2}} \end{array}$$

(2) $$\begin{array}{ccc} PRH & = & \frac{\sum_{f=f_{1H}}^{f_{2H}}{\left|G(f)\right|}^{2}}{\sum_{f=0}^{f_{T}}{\left|G(f)\right|}^{2}} \end{array}$$

where G(f) is the Fourier transform of the signal g(t) and $f_{T}$ is the highest frequency of interest of g(t).

Signals derived from the same event would have similar spectra and then, similar PRL and PRH parameters so, when plotted in a two dimensional map all points would form a packed cluster. Other events may present differences in these parameters, so the clusters would be plotted separately from the first one. Any incoming signal would be analyzed and plotted in the spectral power map in such a way that if it is close to any of the existing clusters it can be classified as events of that type.

The selection of the frequency limits for the intervals is paramount to have separated clusters. This can be done by visual inspection of the spectra of the signals if the differences are notable and there are very few types of events [19]. Otherwise, the classification has to be automatized selecting the intervals according to some criteria and this is precisely what this paper proposes. It seems appropriate that the best set of frequencies would be that which gives the largest separation between clusters.

## 3. Distance Criterion

The points in the spectral power map can be gathered using any clustering technique so we selected the k-means iterative algorithm [20] in the following applications since it is arguably one of the most broadly known clustering algorithms. Notice, however, that it could be replaced by practically any other clustering algorithm since the training data in the case under study is always available with the patterns represented in terms of features *PRH* and *PRL*. For instance, in applications in which the number of clusters is hard to guess from domain knowledge, one could resource to clustering methods such as spectral clustering [21] or graph clustering [22] in which the number of clusters is found in the optimization.

Basically, the process starts defining the number of expected clusters, *k*, and selecting randomly *k* signals as centroids. Then, all the distances between points and centroid are calculated and the events are associated to the nearest cluster. The centroid information is updated using the average positions of all points in the same cluster obtaining *k* new centroids. The process is repeated so some points may change their cluster membership based on their distances to the new centroids. The algorithm ends when a convergence condition is met, the assignment does not change or a maximum number of iterations is reached.

The final goal of the proposed algorithm is to separate signals maximizing the minimum separation between clusters by maximizing the minimum distances between centroids. If $d_{ij}$ is the distance between centroid *i* and *j*, the objective is:

(3) $$D=\max\min_{\begin{array}{c} i\neq j \end{array}}^{k}d_{ij}$$

Additionally, the dispersion of the elements in the clusters has to be considered to calculate *D* in Equation (3), otherwise, very dispersive clusters would have their centroids separated but the points in the clouds may overlap. Then, $d_{ij}$ is defined as a Mahalanobis distance instead of an Euclidean distance:

(4) $$d_{ij}^{2}={\left(C_{i}-C_{j}\right)}^{T}\left(S_{i}^{-1}-S_{j}^{-1}\right)\left(C_{i}-C_{j}\right)$$

being

(5) $$S_{i}=\frac{1}{N_{i}}\sum_{n=1}^{N_{i}}\left(p_{n}-C_{i}\right){\left(p_{n}-C_{i}\right)}^{T}$$

(6) $$S_{j}=\frac{1}{N_{j}}\sum_{n=1}^{N_{j}}\left(p_{n}-C_{j}\right){\left(p_{n}-C_{j}\right)}^{T}$$

where $C_{i}$ and $C_{j}$ are the centroids of clusters *i* and *j*, respectively; and $S_{i}$ and $S_{j}$ are the sample covariance matrices of the elements p in clusters *i* and *j*, respectively. Finally, and $N_{i}$ and $N_{j}$ are the number of elements in cluster *i* and *j*. Using the Mahalanobis distance, the minimum distances between clusters can be maximized and the distances between samples within the same cluster can be reduced.

**Figure 1:**
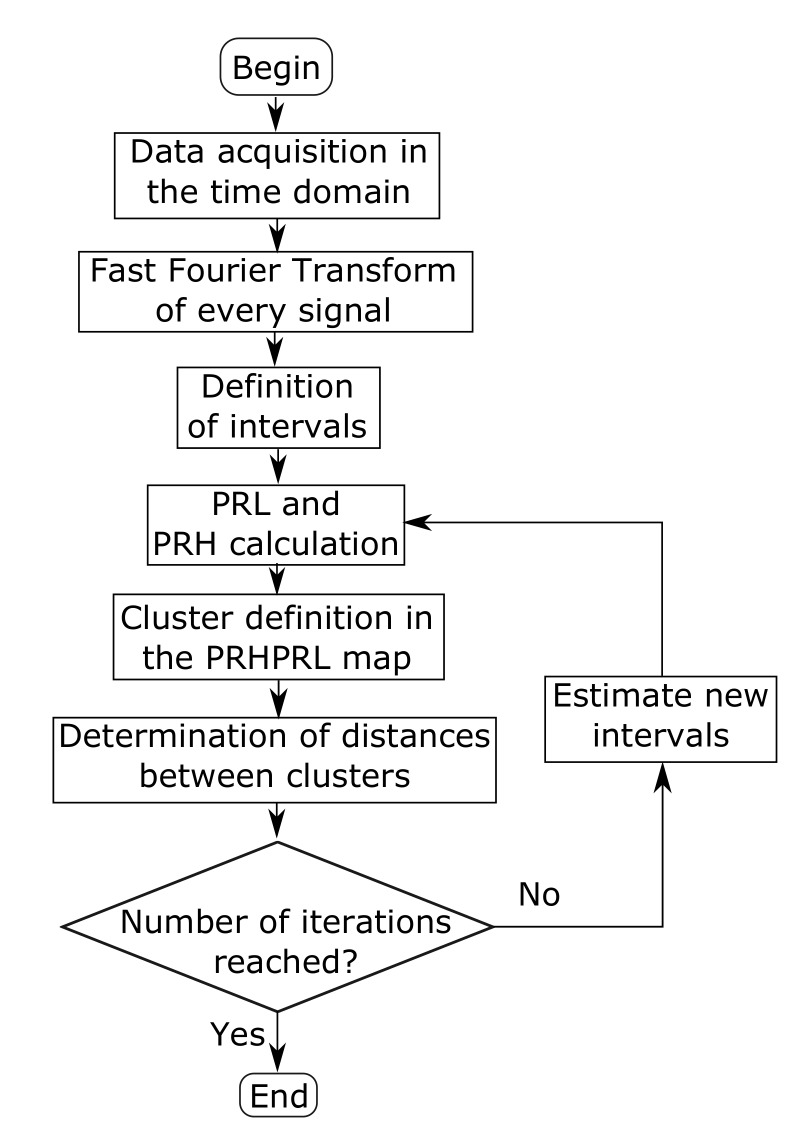
General flow diagram of the algorithm. PRL and PRH are calculated with Equations (1) and (2), respectively.

It is important to remember that the positions in the map represent the spectral power ratios in two bands defined by a set of frequencies $f_{1L}$, $f_{2L}$, $f_{1H}$, $f_{2H}$ and $f_{T}$. Changing these frequencies would move the clusters in the map and would change their shape giving different distances $d_{ij}$ and *D*. Now, the aim is to find the set that maximizes *D*, i.e., to find the most representative bands of frequencies that can differentiate the events. The flow diagram with the steps of the algorithm is represented in Figure 1. The decision to terminate the process is currently based on the number of iterations, though other criteria based on the distances between clusters could be implemented. The maximization of the objective function shown in Equation (3) and represented in the flow diagram as Estimate new intervals, is done with different methods of particle swarm optimization. However, this is not only restricted to PSO since any other optimization method could be used.

## 4. Particle Swarm Optimization

This method places randomly a swarm of entities in the solutions space [23] which in our case has five dimensions defined by the four frequencies of the two bands and the highest frequency $f_{T}$ to give the algorithm the opportunity to select the top frequency of interest for all clusters. In every iteration, every particle is moved around changing its position by the addition of a frequency step, Δf, to all components. Then, the spectral power ratios of Equations (1) and (2) and the distance in Equation (3) are computed for the new intervals. The combination that gives the maximum *D* is stored as the best personal solution for that particle. When the iteration is finished and all particles have moved, the position of the particle with the overall best *D* is stored as the global best. Some constraints have to be supervised during the movement of particles:

- $f_{1L}<f_{2L}\leq f_{1H}<f_{2H}\leq f_{T}$
- Δf has to be multiple of $1/T_{w}$ being $T_{w}$ the sampling window to have exact steps in frequency.
- If any of the frequencies is rendered negative, the particle position is not updated and the speed of the particle is set to naught in order to reduce its inertia.
- If $f_{1L}\geq f_{2L}$ or $f_{2L}>f_{1H}$, $f_{1L}$ and $f_{2L}$ are regenerated randomly considering the first restriction.
- If $f_{1H}\geq f_{2H}$, $f_{1H}$ is regenerated randomly considering the first restriction.

In the next iterations, the movement of the particles is modified by a weighted component that pulls the particle towards its own best and another weighted component that guides the particle towards the global best [24]. The following set of equations represent the original algorithm introduced in [23] and define the position $P_{n}$ and the speed $v_{n}$ of the particle n in every iteration *l*:

(7) $$\begin{array}{ccc} v_{n}\left(l+1\right) & = & v_{n}\left(l\right)+c_{1}U_{1}\left(0,1\right)⊗\left[P_{n,b}\left(l\right)-P_{n}\left(l\right)\right]\\ & + & c_{2}U_{2}\left(0,1\right)⊗\left[P_{b}\left(l\right)-P_{n}\left(l\right)\right],\\ P_{n}\left(l+1\right) & = & P_{n}\left(l\right)+v_{n}\left(l+1\right), \end{array}$$

where $U_{1}\left(0,1\right),U_{2}\left(0,1\right)\in {\left[0,1\right]}^{5}$ are five-dimensional random vectors, with each component independently drawn from a uniform distribution between 0 and 1. Both $U_{1}\left(0,1\right),U_{2}\left(0,1\right)$ randomize the movement of the particles towards their own best $P_{n,b}$ and the swarm’s best $P_{b}$, respectively. The operator ⊗ multiplies the random numbers by the five coordinates component by component. The parameters $c_{1}$ and $c_{2}$ describe the balance between the personal influence of the particle and the social influence in the search of the solution. The original algorithm has been modified in many works to control the convergence towards the global optimum instead of falling in local maxima or minima. Thus, many variants have been proposed to give solutions to different types of problems [25]. In this paper, three approaches to improve the convergence of PSO have been tested with actual measurements: canonical particle swarm optimization [26], time varying inertia weight particle swarm optimization and particle swarm optimization with aging leader and challengers [27].

### 4.1. Canonical Particle Swarm Optimization

In this variation of PSO, the convergence is controlled by a constriction factor, χ, with the idea of exploring in detail the area where a good fitting had been found. This parameter depends on the constants that update the velocity of the particles, $c_{1}$ and $c_{2}$. Then,

(8) $$\chi =\frac{2a}{2-\varphi -\sqrt{\varphi^{2}-4\varphi }},\;\varphi =c_{1}+c_{2},$$

where *a* is a random number between 0 and 1 though it is usually set to 1. The velocity equation is rewritten as:

(9) $$\begin{array}{c} v_{n}\left(l+1\right)=\chi (v_{n}\left(l\right)+c_{1}U_{1}⊗\left[P_{n,b}\left(l\right)-P_{n}\left(l\right)\right]\\ +c_{2}U_{2}⊗\left[P_{b}\left(l\right)-P_{n}\left(l\right)\right]) \end{array}$$

when φ<4 the swarm would attempt to reach the best found solution moving slowly around it while for φ>4 the convergence would be fast and ensured [28]. It is possible to modify the behavior of the swarm choosing different values for $c_{1}$ and $c_{2}$, but, usually, for the sake of simplicity, both parameters are set with the same value. Assuming that φ=4.1 to ensure convergence with $c_{1}=c_{2}=2.05$, the value for χ=0.72984.

### 4.2. Particle Swarm Optimization with Time Varying Inertia

Changing the inertia of the swarm would imprint different velocities to the particles in certain moments when searching for the optimum solution. It is possible to set high velocities when the swarm has to explore large areas of the space of solutions and reduce the speed when some particles had reached their best fittings. This idea was introduced in [29] reducing the coefficient of the inertia from a maximum value $w_{max}$ to a minimum value $w_{min}$ using a linear function (10)

(10) $$w\left(m\right)=w_{max}-\left(w_{max}-w_{min}\right)\frac{m}{M}$$

where w(m) is the coefficient in iteration *m* and *M* is the maximum number of iterations in which the inertia changes its value. The velocity equation is changed into:

(11) $$\begin{array}{c} v_{n}\left(l+1\right)=w\left(m\right)v_{n}\left(l\right)+c_{1}U_{1}⊗\left[P_{n,b}\left(l\right)-P_{n}\left(l\right)\right]\\ +c_{2}U_{2}⊗\left[P_{b}\left(l\right)-P_{n}\left(l\right)\right] \end{array}$$

with M≤L, being *L* the total number of iterations.

### 4.3. Particle Swarm Optimization with Aging Leader and Challengers

Another technique that tries to avoid falling in local maxima or minima is based on giving opportunities to particles different from the best one that could improve the behavior of the swarm. Then, the global best particle $P_{b}$ is the leader of the swarm, $P_{\mathrm{leader}}$, as long as its lifespan is not depleted. The velocity equation is changed into (12). When the leader reaches a certain age, a challenger appears to seize the leadership. This challenger is evaluated during a number of iterations and is accepted as leader if the behavior of the swarm is improved, otherwise, the former leader remains unchanged. The algorithm can be summarized into these steps [27,30]:

(12) $$\begin{array}{c} v_{n}\left(l+1\right)=w\left(m\right)v_{n}\left(l\right)+c_{1}U_{1}⊗\left[P_{n,b}\left(l\right)-P_{n}\left(l\right)\right]\\ +c_{2}U_{2}⊗\left[P_{\mathrm{leader}}\left(l\right)-P_{n}\left(l\right)\right] \end{array}$$

1. Initialization. All particles are randomly deployed in the solution space. The global best particle is selected as the leader, the age, θ is set to 0 and the lifespan Θ to an initial value $\Theta_{0}$.
2. Velocity and position update. All particles are moved according to Equations (12) and $P_{n}\left(l+1\right)=P_{n}\left(l\right)+v_{n}\left(l+1\right)$.
3. Personal best positions and leader $P_{\mathrm{leader}}$ update. If $P_{n}\left(l\right)$ is better than $P_{n,b}\left(l-1\right)$ the personal best for particle *n* is updated. If any of the new positions give a new best solution, the leader is also updated.
4. Lifespan control. Once the positions of all particles have been updated, the age of the leader is increased θ←θ+1 and its lifespan is modified according certain rules shown in Section 4.3.1. If the life of the leader is depleted, θ≥Θ, the algorithm continues in step 5, otherwise, it resumes in step 7.
5. Challenger uprise. A new particle is generated inheriting some coordinates of the leader randomly.
6. Challenger evaluation. The algorithm tests whether the challenger would or would not improve the swarm behaviour during a predefined number of cycles. If the test is positive, the challenger becomes the new leader with an age θ=0 and a lifespan $\Theta =\Theta_{0}$, otherwise, the current leader remains.
7. Check performance. The termination of the algorithm is based on the number of iterations, so this condition checks if l=L. If it is false, the new iteration starts again in step 2.

**Figure 2:**
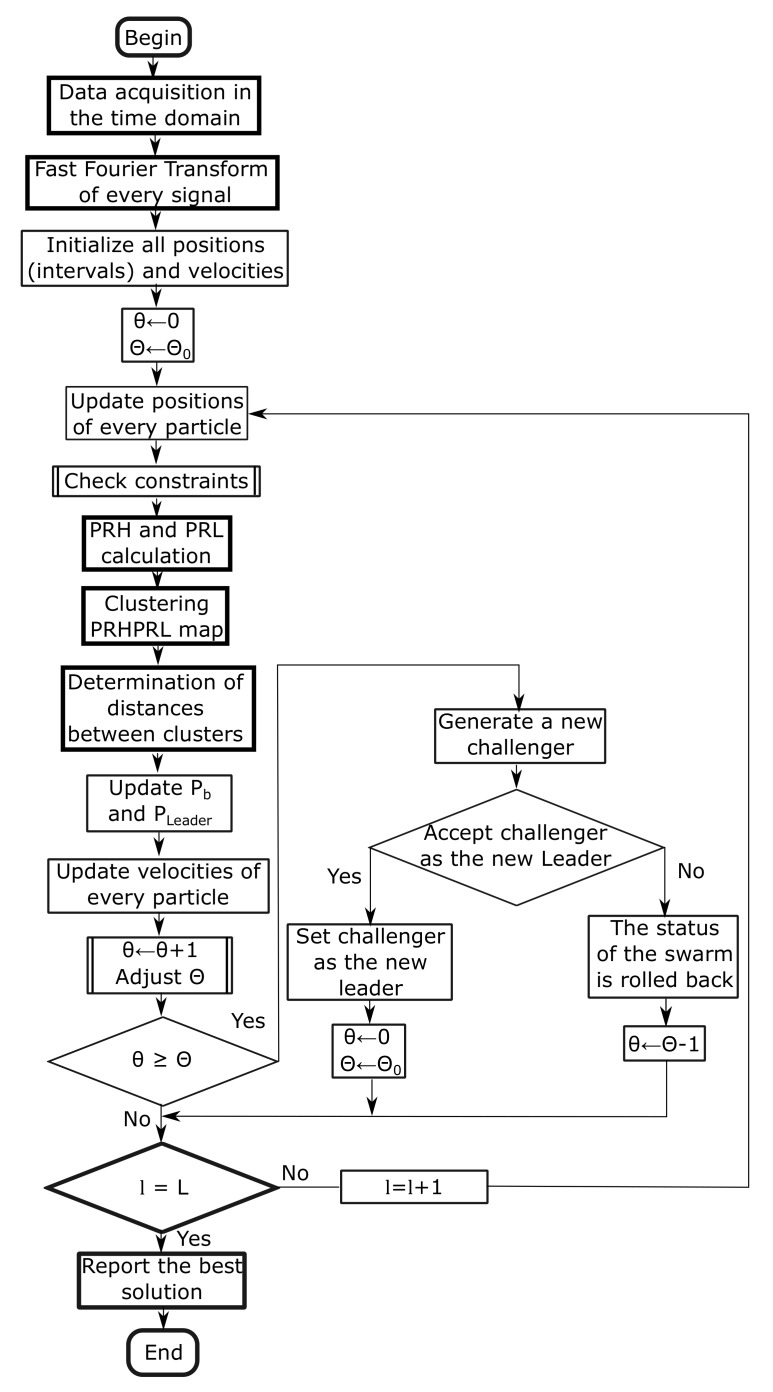
Flow diagram of the particle swarm optimization with aging leader and challengers.

Figure 2 shows the flow diagram of the algorithm with calls to two subroutines to check the constraints of the frequencies in the intervals as formulated at the beginning of this section and to control the lifespan of the leader. The thick line boxes represent the main algorithm described in Figure 1.

#### 4.3.1. Lifespan Control

The rules that define the modification of the lifespan are based on three parameters during the life of the leader: related to the evolution of the global best, $\delta_{P_{b}}\left(\theta \right)$; the change of the personal best solutions accumulated into the parameter $\delta_{P_{n,b}}\left(\theta \right)$ and the evolution of the function for the selected leader, $\delta_{P_{leader}}\left(\theta \right)$, Equation (13).

**Figure 3:**
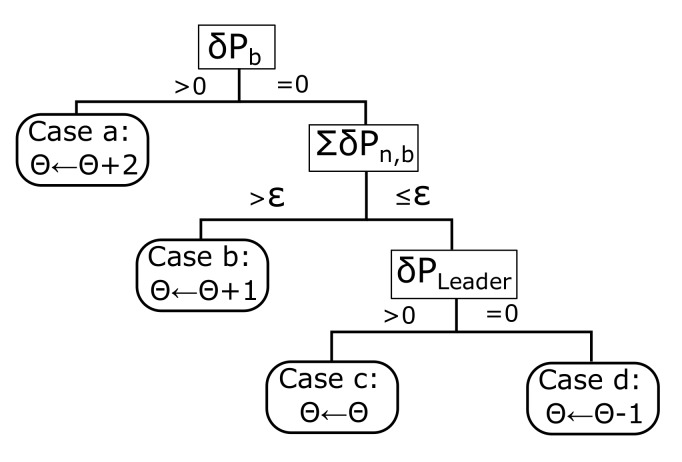
Flow diagram of the lifespan control based on the behavior of the global best, swarm and leader.

All these sequences represent the evolution of the leader and the swarm and evaluate the capability of command of that particle. There are several categories of leadership summarized in Figure 3. Case *a* implies that the leader is capable of improving the global best $\delta_{P_{b}}\left(\theta \right)>0$ guiding the swarm to a better solution so its lifespan is increased in 2. In case *b*, the global best is not improved but the personal bests of the swarm, $\sum_{n=1}^{N}\delta_{P_{n,b}}\left(\theta \right)$ are increased in at least a parameter ϵ, then, the lifespan of the leader is incremented in 1. This parameter ϵ can be adjusted to have more control on the leader’s lifespan considering the achievements of the swarm under his orders. Large values of ϵ would mean a demanding flock while low values would mean that the population is contented with small improvements. We set $\epsilon =0.1\cdot \delta_{P_{b}}\left(\theta \right)$, so the leader can be easily degraded if he does not improve the overall swarm. In case *c*, the only particle that improves is the leader itself $\delta_{P_{leader}}\left(\theta \right)>0$, the decision tree has still confidence in the leader but the lifespan is not modified. Finally, case *d* means that the leader is incapable of improving the former situation and should be changed soon, hence, the lifespan is reduced in 1.

(13) $$\begin{array}{ccc} \delta_{P_{b}}\left(\theta \right) & = & f\left(P_{b}\left(\theta \right)\right)-f\left(P_{b}\left(\theta -1\right)\right)\\ \sum_{n=1}^{N}\delta_{P_{n,b}}\left(\theta \right) & = & \sum_{n=1}^{N}f\left(P_{n,b}\left(\theta \right)\right)-\sum_{n=1}^{N}f\left(P_{n,b}\left(\theta -1\right)\right)\\ \delta_{P_{leader}}\left(\theta \right) & = & f\left(P_{leader}\left(\theta \right)\right)-f\left(P_{leader}\left(\theta -1\right)\right)\\ \theta & = & 1,2,\ldots ,\Theta . \end{array}$$

#### 4.3.2. Challenger Uprise

A challenger appears when the leader is no longer capable of improving the optimization function and its lifespan is exhausted. The challenger inherits some of the coordinates of the leader after a random decision. In our case there is the possibility of changing the low frequency interval, $\left[f_{1L},f_{2L}\right]$ or the high frequency interval $\left[f_{1H},f_{2H}\right]$ plus $f_{T}$. When the decision is taken, another random process generates the frequencies of the chosen interval while the other one remains unchanged.

## 5. Classification of Events

**Figure 4:**
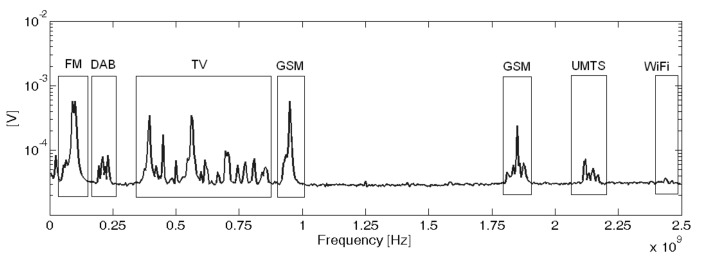
Spectrum of the interferences in the laboratory captured by the antennas without voltage applied to the test objects.

The scope of the algorithm to separate different types of broadband signals gathered in clusters is evaluated in three cases with different frequency ranges. The PSO algorithms presented in Section 4 to maximize the minimum Mahalanobis distance between clusters are tested for the three experiments. The signals in the first two experiments are captured with simple monopole antennas 10 cm long connected directly to a coaxial cable and thence to a high-speed oscilloscope. These antennas are a good option for PD source detection and localization in substations due to their simplicity and omnidirectional radiation pattern [31]. They show special good response below 750 MHz which is suitable for this application since most of the radiation is usually under 600 MHz when the applied AC voltage is high enough to create PD in air [32,33]. Nevertheless, the algorithm would not be affected by the antenna characteristics as long as it has a band suited to the measurement of partial discharges. Therefore the procedure would be able to work with other types of antennas such as logarithmic-periodic, conic, patches or Vivaldi. Moreover, the third experiment was done acquiring the PD with a high-frequency current transformer with a bandwidth in the HF/VHF range to show the ability of the algorithm in separating different types of PD within a phase-resolved PD pattern. The experiments were conducted in an unshielded laboratory so all types of HF/VHF and UHF interferences were affecting the measurements. An example of the spectrum of the radiation received by the antennas without partial discharges can be seen in Figure 4.

Three sets of parameters $c_{1}$ and $c_{2}$ have been used for each PSO method always considering that the individual maxima and global maxima are balanced, so $c_{1}=c_{2}$ for the sake of simplicity. The parameter *a* in Equation (8) is set to 1 and, in the time varying inertia PSO, the number of iterations in which the inertia is modified in Equation (11) is the total number of iterations, M=L. Additionally, the maximum inertia and minimum inertia values are $w_{max}=0.9$ and $v_{min}=0.4$, respectively [29]. All PSO algorithms have been run 20 times with 500 iterations, lifespan Θ=3 and random initialization.

### 5.1. Separating PD Sources in UHF

**Figure 5:**
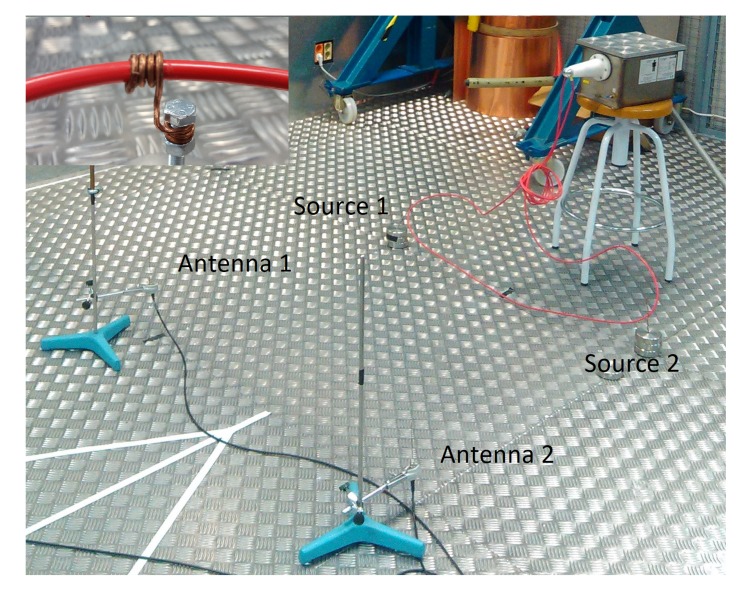
Setup mounted to create two sources of partial discharges with a wound copper wire around a high-voltage cable.

In this experiment, high-voltage is applied to a 20 kV wire that has two separated sections with high divergence electric fields created on purpose for these measurements. Electrical discharges are activated on the surface of the dielectric of the wire and captured with two antennas. Figure 5 shows the setup with two sources of partial discharges in different sections and two antennas that receive the emission. In the top-left of the figure there is a detail of one of the PD sources which consists of a grounded copper wire wound around the high-voltage cable.

The sampling frequency is 5 GS/s and the time window is 1μs, so the frequency step is 1 MHz. Since the times of arrival of the radio-frequency emission of the discharges to the antennas are different, it is possible to know beforehand which pulse corresponds to which section of the wire, [5]. This will be necessary to check whether the classification is correct or not though the information is not used to help the algorithm during the separation of the clusters. In fact, the classification algorithm is only run with the pulses arriving to one of the antennas. The analysis follows the steps explained in Figure 1: First, the parameters PRL and PRH from Equations (1) and (2) are calculated using a random set of frequencies for the intervals. Every event is plotted in a two dimensional map, k-means is used to delimit two clusters and the Mahalanobis distance is calculated with Equation (4). In the next iterations, all three PSO algorithms are applied to maximize the distance changing the set of frequencies that defines the intervals. The results of the worst case scenario, minima of the maxima Mahalanobis distances, are summarized in Table 1 where the ageing leader and challengers PSO (ALC) shows the best behaviour for all cases, specially for $c_{1}=c_{2}=2.05$.

**Figure 6:**
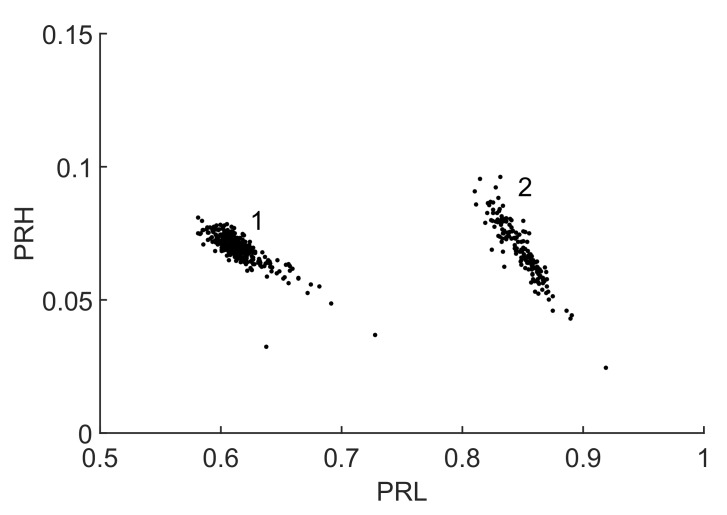
2D map with the clusters of the signals from two sections of the cable emitting partial discharges in the radio-frequency range.

In that case, the resulting clusters are plotted in the 2D map in Figure 6 where they are clearly separated. The selected set of frequencies, $f_{1L}=115$ MHz, $f_{2L}=361$ MHz, $f_{1H}=443$ MHz, $f_{2H}=524$ MHz and $f_{T}=607$ MHz, was found to be the best option to maximize the distance between the two clusters obtaining D=22.28.

**Figure 7:**
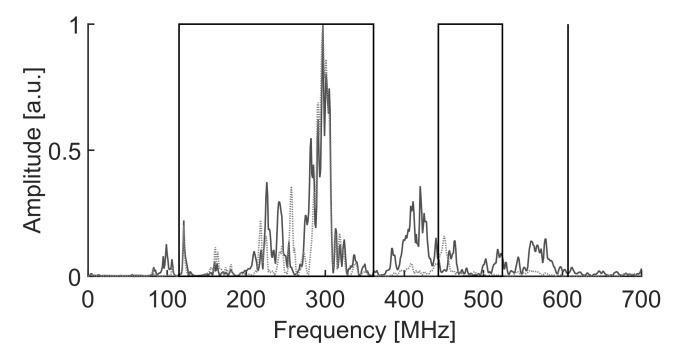
Average spectra of the pulses and the selected intervals that maximize the Mahalanobis distance. The solid line plot corresponds to cluster 1 and the dotted plot to cluster 2.

Figure 7 shows the average spectra of the signals in clusters 1 and 2. Notice that the algorithm does not select the frequencies in the averaged spectra, on the contrary, it analyzes the spectral power of every incoming PD even when the variance is larger. The spectra are plotted in arbitrary units because all components are referred to the peak amplitude. Therefore, low-power signals and high-power signals would be represented equitably. This is done because the signals under study can have different power and eliminating the scale factor helps in the interpretation of what the algorithm is doing. This step does not affect the algorithm since it is a mere representation to explain how the intervals are chosen.

In this particular case, both clusters 1 and 2 have low values for the parameter PRH since most of the power is concentrated inside the low frequency band. Moreover, the selected bands have kept out power of the signals of cluster 1 in the 380–440 MHz and 550–570 MHz bands so the relative power in $\left[f_{1L},f_{2L}\right]$ is lower for this cluster.

**Figure 8:**
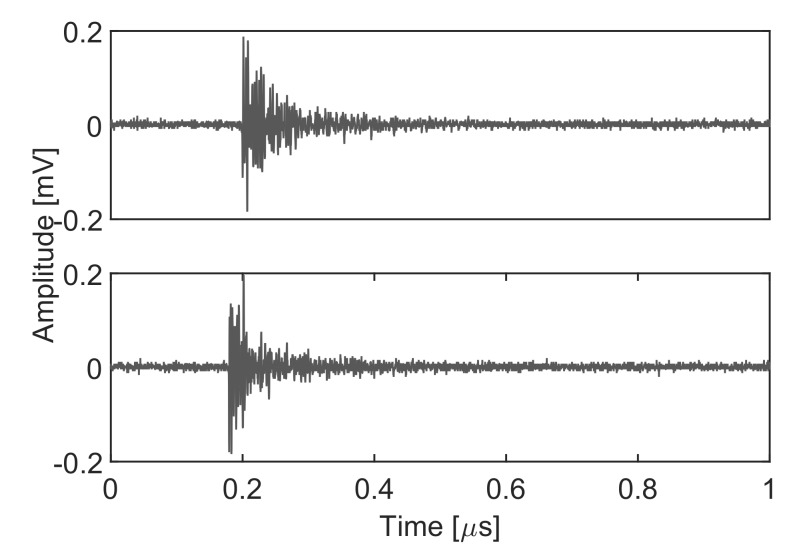
Simultaneous UHF signals from the same type of PD phenomenon.

Any new incoming signal from the wire would be plotted close to any of the clusters. At this stage, the algorithm is capable of separating two partial discharge sources but there is not a correspondence between the clusters and the section of wire that emits the radiation. Using the information of the time of arrival to the antennas of the signals in the clusters, they can be labeled as coming from Section 1 or Section 2 of the wire. Figure 8 shows two examples of PD pulses generated when high-voltage is applied to the wire. The upper plot corresponds to Section 1 and the lower plot to Section 2 of the wire. In summary, despite the fact that both signals are derived from the same pulsed ionization process, this procedure allows for the identification of the origin of the partial discharges with a completely unsupervised algorithm and without any previous training.

### 5.2. Partial Discharges and Radio Interferences

**Figure 9:**
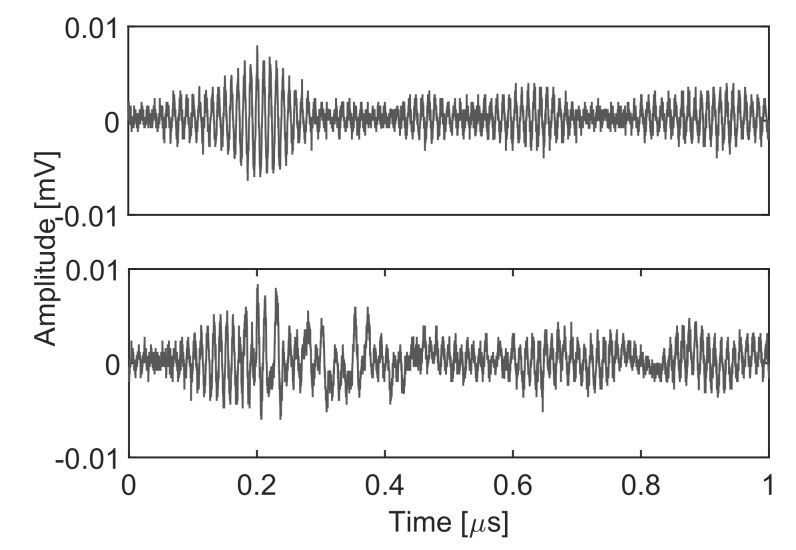
FM radio, upper plot, and partial discharge, lower plot.

In this example, the measurement of partial discharges is disturbed by FM radio signals to show the ability of the algorithm to discern signals with similar levels of energy. These types of disturbances can be very common in the measurement of partial discharges in high voltage overhead lines and substations close to populated areas. In the experiment, the partial discharges are generated in the same way as in the first case winding a grounded wire around a high-voltage cable. The antenna is separated from the source until the emission captured with the antenna is below or close to the level of the radio signal. Figure 9 shows an example of the acquired signals in the time domain with a signal-to-noise ratio (SNR) very close to unity, SNR=1.15. The separation of the clusters defined by the two types of signals is again done maximizing the Mahalanobis distance choosing the intervals with the PSO algorithms. The results shown in Table 2 conclude that the best method would be once more ALC PSO followed very closely by TVI. The best result is achieved with $c_{1}=c_{2}=3$ but it is almost tied with $c_{1}=c_{2}=2.05$.

**Figure 10:**
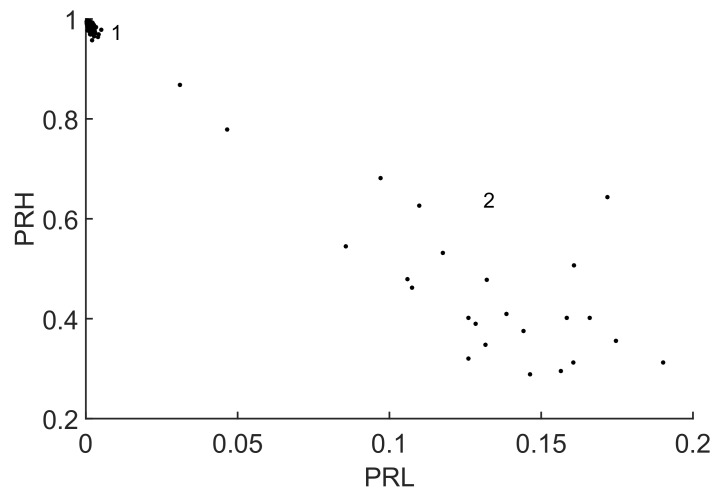
2D map in which cluster 1 corresponds to FM radio and cluster 2 to partial discharges.

**Figure 11:**
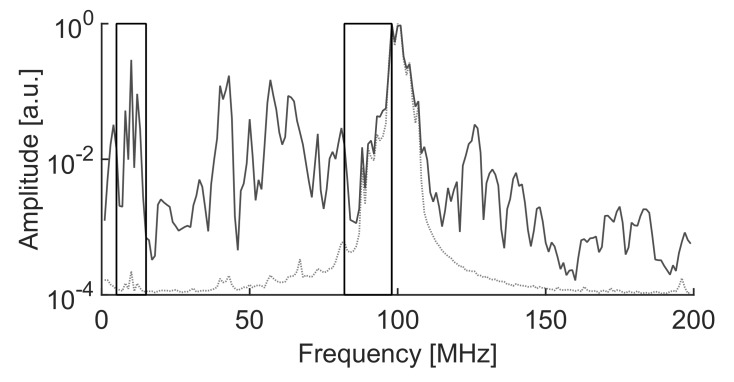
Average spectra of FM signals and partial discharges with the selected intervals. Notice that in this case, the vertical axis is in logarithmic scale to help in the visualization of the spectra. The dotted line corresponds to cluster 1 and the solid line to plot 2 as described in the text.

As a result of the best separation, the selected intervals were $f_{1L}=5$ MHz, $f_{2L}=15$ MHz, $f_{1H}=82$ MHz, $f_{2H}=98$ and $f_{T}=98$ MHz. The time window in this experiment is $T_{w}=1\;\mu$s, so the frequency step is again 1 MHz. The resulting clusters are plotted in a 2D map, Figure 10 and the averaged spectra of the signals are plotted in Figure 11.

Notice that the algorithm has decided to discard the information above $f_{T}=98$ MHz where both signals still have power. Then, it selects the low frequency interval, $\left[f_{1L},f_{2L}\right]$, where the power of the signal with solid line is quite high and the power of the signal with dotted line is very low. This places cluster 2 to the right of the map, high PRL, and cluster 1 to the left, low PRL. Finally, the ALC PSO algorithm chooses the high frequency interval where the power of the signal with dotted line has most of the power so pushes cluster 1 to the top of the map, highest PRH. Cluster 2 has more power in the high frequency band than in the low frequency band so the PRH is relatively high with high dispersion.

The identification of the signals is easy because the partial discharges have power in frequency bands below the minimum FM radio frequency in 87.5 MHz and above 108 MHz, solid line, whereas the FM radio spectrum is strictly confined in the 87.5–108 MHz band, dotted line, resulting from the smooth modulated signals shown in Figure 9. Moreover, signals in cluster 2 are very scattered since PD have a stochastic nature and, hence, the spectral characteristics are not uniform. To assert these statements, taking a sample of a signal already classified in one of the clusters and analyzing its spectrum would label that cluster as FM radio or partial discharge.

### 5.3. High Frequency and Very High Frequency Signals

The nature of the partial discharges used in this case is completely different to demonstrate the capability of the algorithm in separating any type of PD. Now, the measuring setup is in agreement with the indirect detection circuit of the IEEE 1434 guide [34] including a coupling capacitor in parallel with the test objects to provide a low-impedance path for the PD. This also complies simultaneously with the standard IEC-60270 [35] for the identification of PD sources. The pulses and noise are acquired with a high frequency current transformer in the bands of frequency spanning from the high frequency range (HF) to the very high frequency range (VHF). This setup allows separating pulses using the wideband characteristic of the sensor and, in addition, identifying sources with the phase-resolved partial discharge (PRPD) patterns. The proposed separation technique has been extended to this frequency range to confirm the correct separation of the physical phenomena occurring in the insulations with the help of these PRPD patterns. Apart from noise, there are two types of signals occurring simultaneously: partial discharges occurring inside a 300 kVA power transformer and corona discharges due to the ionization of a sharp point at high-voltage. An inductive sensor captures all signals together and the algorithm is capable of classifying them in three clusters maximizing their distances. A total set of 2948 events were acquired during several minutes of simultaneous activity of electrical discharges and noise. The results are summarized in Table 3 where the ALC PSO shows again the best behaviour for all $c_{1}=c_{2}$ parameters.

**Figure 12:**
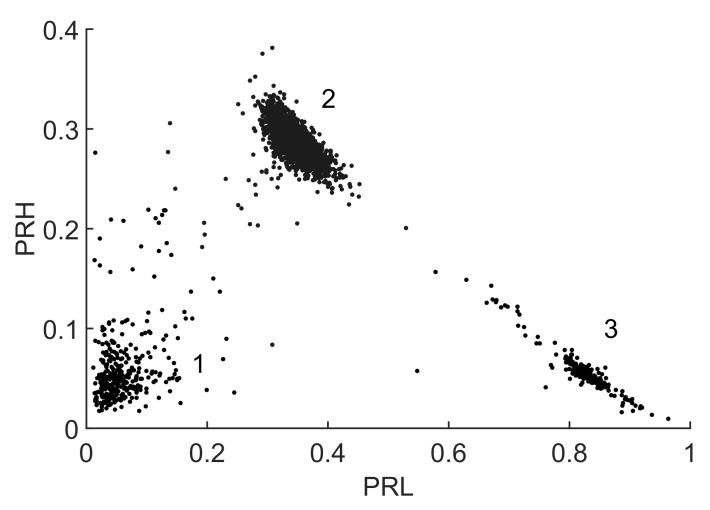
2D map of the three separated clusters corresponding to the VHF experiment.

**Figure 13:**
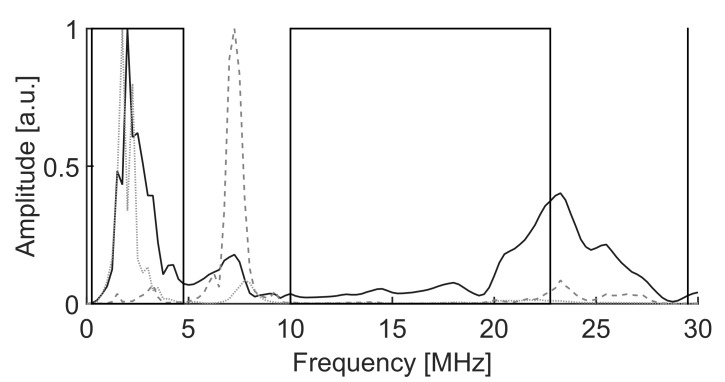
Spectra of the three types of signals. The dashed line corresponds to the average spectra of cluster 1, the solid line corresponds to cluster 2 and the dotted line to cluster 3.

Figure 12 shows the classification of the three types of signals in clusters using the selected intervals to maximize the Mahalanobis distance where $f_{1L}=0.25$ MHz, $f_{2L}=4.75$ MHz, $f_{1H}=10$ MHz, $f_{2H}=22.75$ MHz and $f_{T}=29.5$ MHz. The sampling frequency is $f_{s}=200$ MS/s, the time window is now 4μs so the frequency step is 250 kHz. The algorithm considers that the information from $f_{T}=29.5$ MHz to $f_{s}/2=100$ MHz is irrelevant to separate the clusters. The averaged spectra of each cluster are shown in Figure 13. Observing the intervals, it can be seen that the dotted spectrum falls almost completely inside the low frequency interval, so those signals will be plotted with the highest PRL which corresponds to cluster 3 in Figure 12. Most of the power in the dashed line spectrum is left out of the selected bands, so it will be plotted with low PRL and PRH, corresponding to cluster 1 in Figure 12. Finally, the solid line spectrum has power in both intervals so it will be plotted in the center of the map, corresponding to cluster 2 in Figure 12.

**Figure 14:**
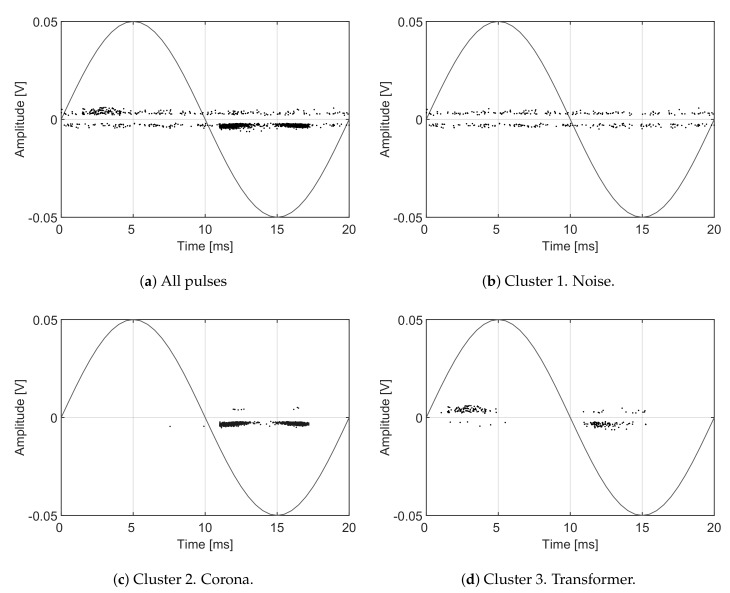
Phase-resolved patterns to identify the type of pulses.

**Figure 15:**
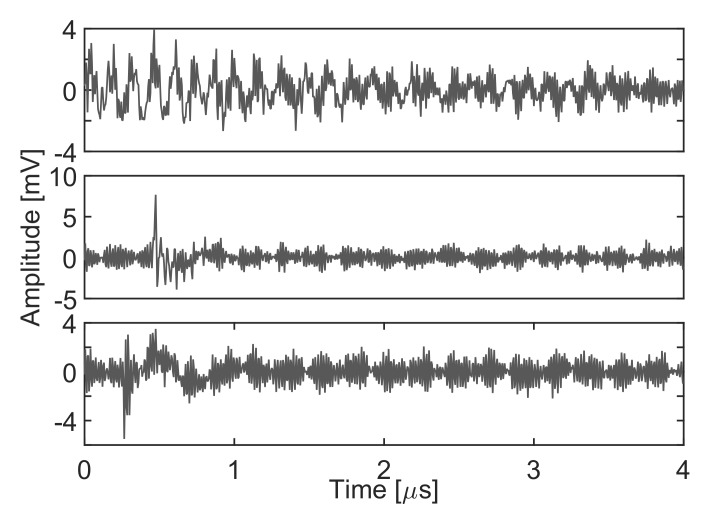
Three types of signals. The upper plot is electrical noise induced and conducted through the wire; the middle plot is one type of pulse corresponding to corona discharges and the lower plot is a pulse derived from discharges in the transformer.

As mentioned before, the algorithm does not identify the type of signal which should be done with additional information. Thus, at the moment, the only information we have is which signals and spectra correspond to which cluster but the origin of the signals is unknown. In this particular case, the nature of the electrical discharges can be known using a phase-resolved PD pattern, [36]. A high-frequency current transformer connected to the wire to ground that conducts the pulses would capture the signals shown in the pattern of Figure 14a where all dots are mingled together and it is difficult to identify their origin. Applying the separation algorithm, the events can be classified into three clusters. Then, selecting the elements of every cloud and plotting the patterns using the phase information [36], it is possible to obtain the rest of plots in Figure 14. Particularly, Figure 14b corresponds to cluster 1 in Figure 12 and it is identified as noise because the pattern is not correlated with the voltage phase. Figure 14c corresponds to cluster 2 which is identified as corona discharges because they occur only in one semi-cycle of the applied voltage. Finally, Figure 14d corresponds to cluster 3 which are internal discharges in the transformer because they occur in both semi-cycles and close to the zero-crossings of the voltage. Figure 15 shows an example of the three types of pulses. Notice that, though the signal-to-noise ratio is pretty poor, the algorithm is again able to separate the different types of signals.

There are dots in the map in Figure 12 that are not close to any of the clouds and could be discarded. The k-means clustering algorithm used in these examples associates all points to a certain group so, even when those events can be considered outliers they are assigned to a cluster. They also appear in Figure 14c,d and can be identified as dots with different voltage sign than those of the packed groups; they are, in fact, noise.

## 6. Conclusions

The application of PSO to enhance the performance of the PR separation technique has been deeply studied in this paper. Three different PSO algorithms have been compared to separate the clusters in the most efficient way leading to good results. The method can be applied to the classification of any type of signal as long as the information of interest is found in its spectral characteristics and it has been tested with partial discharges measured in the UHF and HF/VHF bands. Unlike other feature extraction techniques, the nature of the signal and the physical meaning of the outcome solution is preserved so further deductions on the results can be conducted. The spectral characterization has been limited to two bands of frequency to present clear and intuitive clusters in two dimension plots. The k-means clustering technique has been found to be suitable in the tested cases though it can be further improved by including more sophisticated methods where it is not necessary to know the number of classes a priori or the borders of the clouds are better defined such as spectral clustering. In every iteration, once the clusters have been defined, three different PSO algorithms are tested to find the bands of frequencies that maximize the minimum separation between clusters, and consequently, the bands of frequencies where the classes have more differences in their spectra. Thus, the proposed objective function calculates the distance between the centroids of the clusters, and maximizes the minimum distance in every iteration. Other approaches can be followed such as to maximize the sum of the distances between clusters or maximize the area of a polygon whose vertices are the centroids. The distance is another variable in the method that can be tweaked. Currently it is defined as the Mahalanobis distance to consider the dispersion of data inside the cluster. However, the method is open to the use of other similarity measures that capture prior knowledge about the problem. During the process with the PSO algorithms, it has been found that the method is very sensitive to the selected components of the frequency intervals because the power spectra of PD are usually very spiked. This means that changing one of the components of the frequency bands can move the positions of the clusters in the map from one place to another one far away. The result is that the separations achieved by the PSO algorithms are not the same every time they are run. Nevertheless, in the examples, after running all PSO 20 times, all the frequency bands selected were those that gave the worst distances between clusters and, hence, the poorest separation of the signals. Even in the worst case scenario, those bands were sufficient to have the clusters clearly identified and separated. The ALC PSO method achieves good results when the parameters $c_{1}$ and $c_{2}$ are low because it is capable of exploring larger areas of the solutions space thanks to the change of leader. The other PSO algorithms, CAN and TVI, may have early convergence at a relative maximum where they remain stuck because they do not have challengers which allow to explore new regions. Increasing $c_{1}$ and $c_{2}$ gives more mobility to the particles improving the performance of TVI to reach distances very similar to those obtained with ALC. However, the type of PSO is not critical and simpler PSO algorithms could also be used to separate the different types of signals or even other optimization methods. Moreover, considering the execution times for 500 iterations for each method and experiment in Table 4, the ALC PSO is clearly the slowest so, if computing time is an important constraint, any other method would be recommended.

## Figures and Tables

**Table 1:** Minima of the maxima Mahalanobis distances achieved with Canonical PSO (CAN), time-varying inertia PSO (TVI) and ageing leader and challengers PSO (ALC) in the case of UHF signals.

	$c_{1}=c_{2}=1$	$c_{1}=c_{2}=2.05$	$c_{1}=c_{2}=3$
CAN	17.62	17.93	16.95
TVI	12.24	14.49	19.79
ALC	**18.20**	**22.28**	**21.36**

**Table 2:** Mahalanobis distance achieved with Canonical PSO (CAN), time-varying inertia PSO (TVI) and ageing leader and challengers PSO (ALC) for the experiment with partial discharges and FM radio disturbances

	$c_{1}=c_{2}=1$	$c_{1}=c_{2}=2.05$	$c_{1}=c_{2}=3$
CAN	22.18	22.12	12.33
TVI	8.78	32.26	29.54
ALC	**22.36**	**33.60**	**33.65**

**Table 3:** Minima of the maxima Mahalanobis distances achieved with Canonical PSO (CAN), time varying inertia PSO (TVI) and ageing leader and challengers PSO (ALC) for the experiment with very high frequency signals.

	$c_{1}=c_{2}=1$	$c_{1}=c_{2}=2.05$	$c_{1}=c_{2}=3$
CAN	8.70	8.65	8.79
TVI	9.07	9.91	13.62
ALC	**15.68**	**16.33**	**16.07**

**Table 4:** Execution times in minutes for 500 iterations for every PSO method and experiment.

	CAN	TVI	ALC
UHF	3.13	2.23	38.27
HF/VHF	8.50	7.78	31.16
FM	2.47	3.79	32.14
